# Add-on multiple submucosal injections of the RNA oligonucleotide GUT-1 to anti-TNF antibody treatment in patients with moderate-to-severe ulcerative colitis: an open-label, proof-of concept study

**DOI:** 10.1186/s41232-024-00332-7

**Published:** 2024-04-25

**Authors:** Kenji Suzuki, Yukinori Sameshima, Junji Yokoyama, Shuji Terai, Hiroyuki Yoneyama, Raja Atreya, Markus F. Neurath, Toshifumi Hibi, Hitoshi Asakura

**Affiliations:** 1 Department of Gastroenterology, Niigata University Medical and Dental Hospital, 2-746 Asahimach-Dori, Chuo-Ku, Niigata-Shi, Niigata, 951-8518 Japan; 2 Department of Health Informatics, Niigata University of Health and Welfare, 1398 Shimami-Cho, Kita-Ku, Niigata-Shi, Niigata, 950-3198 Japan; 3 Sameshima Hospital, 9-8 Kajiya-Cho, Kagoshima-Shi, Kagoshima, 892-0846 Japan; 4 GUT Inc, 4563-1 Niida, Kochi-Shi, Kochi, 781-0112 Japan; 5 Department of Medicine 1, University Hospital Erlangen, Friedrich-Alexander-Universität Erlangen-Nürnberg, Ulmenweg 18, 90154 Erlangen, Germany; 6 Center for Advanced IBD Research and Treatment, Kitasato Institute Hospital, Kitasato University, 5-9-1 Shirokane, Minato-Ku, Tokyo, 108-8642 Japan

**Keywords:** Ulcerative colitis, CHST15, TNF antibody, Endoscopic improvement, Clinical remission

## Abstract

**Background:**

Carbohydrate sulfotransferase 15 (CHST15) is an enzyme biosynthesizing matrix glycosaminoglycan that modulates tissue remodeling. We evaluated the efficacy of add-on submucosal injections of GUT-1, the RNA oligonucleotide inhibitor of CHST15, to ongoing anti-tumor necrosis factor (TNF) antibody treatment in patients with moderate-to-severe ulcerative colitis (UC).

**Methods:**

This was an open-label study of 250 nM of GUT-1 by endoscopic submucosal injections at weeks 0, 2, 4 in five UC patients who lost response during maintenance treatment to anti-TNF antibodies. The primary endpoint was the rate of endoscopic improvement at week 6 and secondary endpoints included the rates of clinical remission by modified Mayo Score (mMS). Patients received follow-up observation with continuous maintenance treatment by the same anti-TNF antibody till the time of clinical recurrence or for overall 52 weeks.

**Results:**

At week 6, rates of endoscopic improvement and clinical remission were 80% (*n* = 4/5) and 60% (*n* = 3/5), respectively. The mean Endoscopy Subscore was reduced from 2.4 (95%CI: 1.7 to 3.1) at baseline, to 1.0 (95%CI: 0.1 to 1.9) at week 6. The mean mMS was reduced from 7.8 (95%CI: 6.2 to 9.4) to 1.3 (95%CI: 2.9 to 4.3). GUT-1 was well tolerated. Three patients did not show clinical recurrence for 52 weeks. All three corticosteroid-dependent patients showed no corticosteroid exposure for at least 24 weeks after achieving clinical remission. Multiple dosing was also well tolerated.

**Conclusions:**

Add-on multiple injections of GUT-1 to ongoing anti-TNF antibody was able to induce rapid and durable clinical responses in UC patients who lost response to anti-TNF therapy.

**Trial registration:**

Clinical trial Registration Number (Japan): UMIN000020900.

## Background

Progressive bowel destruction causes refractory conditions against available anti-inflammatory treatment options in inflammatory bowel disease (IBD) [1–5]. Structural bowel damage includes refractory ulcerations, onset of fibrosis and fistula formation, which all are manifestations of mucosal tissure remodeling [3–5]. Mucosal tissue remodeling in IBD leads to unfavorable clinical outcomes such as surgery and is thus an emerging and important therapeutic target. We have here focused on extracellulat matrix (ECM) molecules, especially carbohydrate sulfotransferase 15 (CHST15), as a potential novel therapeutic target in IBD [6, 7]. CHST15 is a type II transmembrane Golgi protein that biosynthesizes highly sulfated disaccharide units (E-units) of chondroitin sulfate (CS), which binds to various functional proteins and pathogenic microorganisms [6–8]. Matrix CS-E is shown to activate fibroblasts through Wnt and receptor for advanced glycation end product (RAGE) signaling pathways and to promote collagen fibril formation [6–9]. In several disease models, blockade of CHST15 prevented or repressed fibrosis formation in esophageal strictures, experimental colitis, cardiac and pulmonary fibrosis [9–13].

GUT-1 is a synthesized double stranded RNA oligonucleotide that selectively inhibits the expression of the CHST15 gene through siRNA mechanisms [9, 14, 15]. CHST15 siRNA suppressed CHST15 mRNA in a dose dependent manner and demonstrated repression of fibrosis-related genes in human fibroblast cell lines [11, 13]. In a Phase I trial, a single submucosal injection of GUT-1 was well tolerated and showed endoscopic improvement in patients with Crohn’s disease (CD) who did not respond to conventional treatment [15]. In histology assessments, reduction of the target CHST15 protein and repression of pre-established fibrosis were shown, suggesting the improvement of bowel tissue remodeling [9, 15]. In a phase IIa induction trial, one-time submucosal injection of 250 nM of GUT-1 demonstrated 71.4% of endoscopic improvement (vs. 28.6% for placebo) and 57.1% of clinical remission (vs. 14.3% for placebo) at week 4 in patients with ulcerative colitis (UC) refractory to conventional treatment [16]. In histological evaluation, reduction of mucosal target CHST15 protein expression and reversal of tissue remodeling was shown [16]. Thus, GUT-1’s action in improving tissue remodeling was suggested also in patients with IBD [9, 17].

Anti-tissue remodeling agents have distinct mechanisms in comparison to advanced biological therapies, thus they are expected to complement the therapeutic efficacy of biologics, especially in patients with secondary failures. To investigate whether add-on anti-tissue remodeling therapy induces clinical response in UC patients who insufficiently responded to anti-TNF antibody treatment and maintained moderately-to-severely active disease activity, we performed an investigator-initiated trial (IIT) of multiple submucosal injections of GUT-1, as add-on therapy to ongoing anti-TNF antibody treatment, as a 6-week induction regimen. The patients continued to receive maintenance therapy by anti-TNF antibody after stopping GUT-1 add-on treatment and were observed till the time of clinical recurrence or for overall 52 weeks.

## Methods

### Study design and participants

This was an open-label, investigator-initiated, monocentric-study which included 5 adult patients (age 16–75 years) with moderate-to-severe UC defined by a modified Mayo Score (mMS) of 5 to 9 and a Mayo Endoscopy Subscore of ≥ 2 (Table 1) [18–20]. The induction study was conducted from February 2016 to April 2016 in Sameshima Hospital in Japan. Patients had to be previously treated for more than 3 months before the screening tests by anti-TNF antibodies (infliximab: IFX or adalimumab: ADA) and experienced an insufficient response to ongoing anti-TNF therapy confirmed by clinical and endoscopic activity.

**Table 1:** Demographic and baseline characteristics

	GUT-1 (*n* = 5)
Male (n)	4
Female (n)	1
Mean age (years)	38.2
Mean duration of disease (years)	5.8
Mean Endoscopy Subscore	2.4
UC location: n (%)	
Left-sided colitis	5 (100)
Mean modified Mayo Score (mMS)	7.8
Anti-TNF antibodies: n (%)	
Infliximab (IFX)	3 (60)
Adalimumab (ADA)	2 (40)
Refractory to prior anti-TNF antibody: n (%)	5 (100)
Concomitant anti-TNF antibody: n (%)	5 (100)
Concomitant corticosteroid: n (%)	3 (60.0)
Concomitant thiopurine: n (%)	3 (60.0)
Concomitant tacrolimus: n (%)	2 (40.0)
Concomitant 5-ASA: n (%)	5 (100)

Exclusion criteria were as follows: (i) History of serious cardiac, hematological or pulmonary disease; (ii) History of complete colon resection surgery; (iii) A hepatic impairment or renal disorder; (iv) History of malignant tumor within the past 5 years; (v) A complication of serious infection that requires hospitalization; (vi) History of clinically serious allergic symptom; (vii) Positive for HBs antigen and HCV antibody; (viii) Alcohol or drug dependency; (ix) Received any other investigational product within 6 months prior to the informed consent; (x) Psychiatric or neurological disorder; (xi) Unsuitable for participation for any other reason; (xii) Incapable of or restricted to the protocol-directed examinations or procedures: (xiii) Not willing and able to use a contraceptive method, which the investigator considers reliable; (xix) Pregnancy or lactation for females. Written informed consent was provided by all patients prior to the inclusion into the study.

### Procedure

**Fig. 1:**
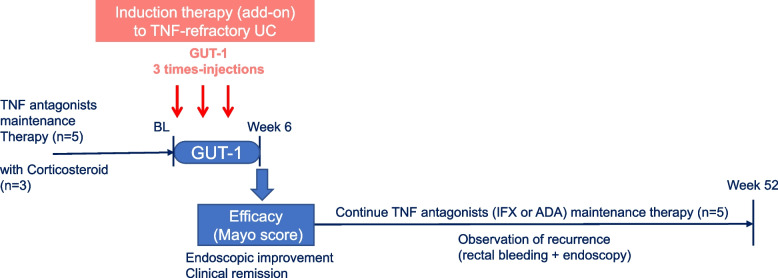
Clinical study design. Five subjects were screened, and none of them were screen failures. Five subjects were enrolled in the study and received 250 nM of GUT-1 at week 0 (baseline), 2 and 4. Endoscopy was done in all patients at week 0, 2, 4 and 6 for induction regimen and at appropriate time points during follow-up period for up to 52 weeks

Patients received 3-times submucosal injections of 250 nM of GUT-1 (synthesized by BioSpring, Frankfurt, Germany) at 2 weeks-interval [week 0, 2 and 4] via endoscopy (Fig. 1). The dose, total number of injections and dosing interval were determined based on our previous pharmacokinetics and safety results from Phase I clinical trial, and nonclinical toxicology results from repeated dose toxicity studies in cynomolgus monkey [11, 15]. GUT-1 was administered submucosally (2 mL/site by one injection and 2-site injections at distances of 35 cm, 25 cm, 15 cm and 5 cm from the anal verge, thus, 8 site-injections in total and 16 mL in total volume of injection) during the ongoing endoscopic procedure [9]. Eligible subjects visited to the study site on the dates (week 0, 2, 4) of GUT-1 administration. They returned to the study site for follow-up examinations at week 6 and for long-term observation thereafter for 52 weeks at the maximum (Fig. 1). For endoscopic examinations between weeks 0 (baseline: BL), 2, 4, and 6, photographs were taken from the same sites of GUT-1 administration and sites of heaviest inflammation. Biopsy was taken from the heaviest lesion at baseline. Between weeks 0 (baseline) and 6, all biopsies were then taken from the same sites.

### Outcome measures

The primary endpoint was the rate of endoscopic improvement at week 6. Endoscopic improvement was defined as a Mayo Endoscopy Subscore of 0 or 1 [18–20]. Secondary endpoints included clinical responses and clinical remission. Clinical response was defined as a decrease from baseline in the mMS of greater than or equal to 2 points and at least a 30% reduction from baseline, and a decrease in RBS of greater than or equal to 1 or an absolute RBS of 0 or 1. Clinical remission was defined as a mMS score of 0 to 2, including the following 3 components; 1) Stool Frequency Subscore (SFS) of 0 or 1, 2) Rectal Bleeding Subscore (RBS) of 0, and 3) Endoscopy Subscore of 0 or 1 [18–20]. The percentage changes in the mMS, SFS, RBS and Endoscopy Subscore from baseline to week 2, 4 and 6 were also evaluated. Histological response was exploratory evaluated by the Nancy histological Index (NHI) [21–23] at BL and week 6. Histological improvement was defined as a ≧1-point reduction in the NHI from baseline [23]. Histological assessment was performed by 2 independent pathologists, blinded to all data. In the case of discrepancy between pathologists, the worst score was taken.

Safety data (incidence and type of AEs; markedly abnormal changes in laboratory values and vital signs and ECG) of all treated subjects was collected from the day of consent obtainment to week 6 after the first administration of GUT-1. The incidence and the type of AEs were summarized by treatment groups. For histological analysis, biopsy specimens were fixed in 4% formalin, embedded in paraffin and were stained with HE methods.

### Follow-up observation

After stopping add-on GUT-1 treatment, the patients continued to recive unchanged (dose, frequency) ongoing anti-TNF antibody treatment as a strandard maintenance therapy regimen. All patients underwent follow-up observation till the time of clinical recurrence or 52 weeks if there was no sign of clinical recurrence. The sign of clinical recurrence was noticed by occurrence of patient-reported rectal bleeding and the recurrence was subsequently confirmed by endoscopic examination. Once clinical recurrence was confirmed, the investigators changed treatment by escalating the ongoing anti-TNF antibody therapy with dose intensification, initiating corticosteroid treatment or initiation of another anti-inflammatory therapy. Follow-up endoscopy was also performed at least once within 6-months, even if there was no sign of clinical recurrence during the observation period.

### Data analysis and statistical methods

Demographic and other baseline characteristics were summarized by tabulating frequency. Descriptive statistical analysis was performed using GraphPad Prism version 9. mMS and NHI were calculated for each subject, and % changes from baseline for mMS and its 3 components, MES, SFS, RBS were calculated. These scores were tabulated in by-subject listings. The proportions of participants with endoscopic improvement, clinical remission and histological improvement were calculated. The results of the experiments are expressed as mean ± 95% CI. The main focus was on complete descriptive statistics.

### Ethical statement

The study was performed after prior informed consent from all subjects and under a protocol that was approved by the responsible Ethics Committee, and was conducted in accordance with the Declaration of Helsinki and its later amendments. Prior to initiating the clinical trial, Clinical Trial Notification was registered in the University Hospital Medical Information Network (UMIN) Clinical Trials Registry (UMIN000020900). Registered 8 February, 2016 (https://center6.umin.ac.jp/cgi-open-bin/ctr_e/ctr_view.cgi?recptno=R000024112).

## Results

### Patient population and baseline characteristics

A total of 5 UC patients were enrolled into the study. There was no screrening failure. All patients showed left-sided colitis with moderate-to-severe disease. The percentage of patients who received ongoing anti-TNF antibody (3 subjects for IFX at a dose of 5 mg/kg with 8 week-interval, or 2 subjects for ADA at a dose of 40 mg with 2 week-interval) treatment for over three months was 100% (Table 1) and all were judged as anti-TNF antibody-refractory due to confirmed ongoing clinical and endoscopic activity. Concomitant medication with azathioprine was used by 60% (3/5). Concomitant medication with tacrolimus was used by 40% (2/5). Three of 5 patients (60%) showed corticosteroid-dependency and received oral corticosteroids as concomitant medication (40 mg for case 1 and 20 mg for cases 4 and 5, Table 2). Corticosteroid dose was stable for 2 weeks prior application of GUT-1. There was no dose intensification of corticosteroid therapy throughout the study. All subjects received oral 5-aminosalicylic acid (5-ASA) therapy as a baseline concomitant medication in unchanged dose 14 days prior GUT-1 application and throughout the study. Three subjects received oral azathioprine (50 mg/day) in unchanged dose throughout the study. Two subjects received oral tacrolimus (0.1 mg/kg by adapting trough whole-blood levels of 5 to 10 ng/mL) in unchanged dose during the induction study period. There was no discontinuation.

**Table 2:** Individual summary

Case	Age/Sex	DD	DD^a^-TNF	CM	Modified Mayo Score					RS W6	RM W6	EI W6	Recurrence W52
						BL	W2	W4	W6				
1	23F	1	0.6 IFX	ADA, AZP, PSL, 5-ASA	ES	2	2	1	0	✔	✔	✔	(-)
					mMS	8	7	3	0				
2	51 M	12	6 IFX	ADA, TAC, 5-ASA	ES	2	1	1	1	✔		✔	W44 ( +)^b^
					mMS	6	3	2	2				
3	35 M	7	4 IFX	IFX, AZP, 5-ASA	ES	2	2	2	1	✔	✔	✔	(-)
					mMS	7	4	3	2				
4	32 M	4	4 IFX	IFX, PSL, 5-ASA	ES	3	2	1	1	✔	✔	✔	W36 ( +)^b^
					mMS	9	7	3	2				
5	50 M	5	4 IFX	IFX, AZP, PSL, TAC, 5-ASA	ES	3	2	2	2	✔			(-)
					mMS	9	3	3	3				

*DD* Duration of disease (year), *CM* Concomitant medication, *ADA* Adalimumab, *AZP* Azathioprine, *PSL* prednisolone, *ASA*, *IFX* Infliximab, *TAC* Tacrolimus, *ES* Endoscopy Subscore, *mMS* Modified Mayo Score, *BL* Baseline, *W* Week, *RS* Clinical response, *RM* Clinical remission, *EI* Endoscopic Improvement. ✔ Achieved RS, RM, EI at week 6

^a^DD-TNF: Duration of disease (year) that exposed to prior anti-TNF antibody (IFX) before this study. In Case 1 and case 2, IFX was changed to ADA because of no response to IFX in the history and received ADA at least for 3 months before screening

^b^Clinical recurrence was confirmed by rectal bleeding and endoscopic examination (Week 44 for case 2 and week 36 for case 4) before the end of observation (Week 52)

### Efficacy in endoscopic responses

**Fig. 2:**
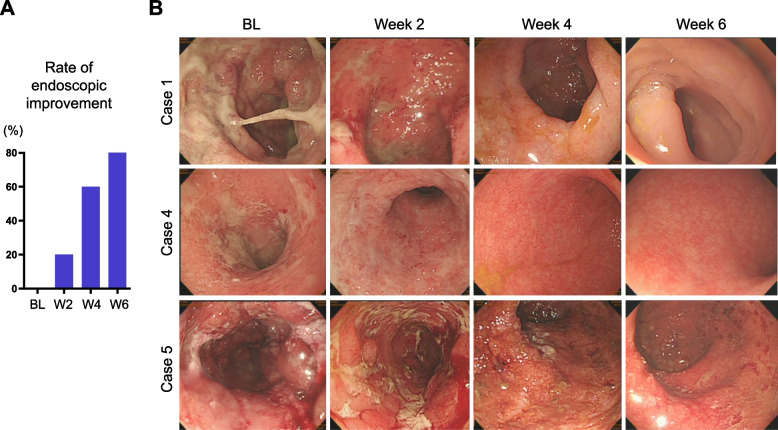
Induction of endoscopic improvement by add-on GUT-1. **A** Rates of endoscopic improvement at baseline (BL), week 2, 4 and 6. **B** Endoscopic findings of the heaviest lesion at baseline (BL; week 0) and the same lesion at weeks 2, 4 and 6 in 3 corticosteroid-dependent UC patients are presented (case 1, 4 and 5)

At baseline (week 0), all patients showed moderate or severe disease activity in the Mayo Endoscopy Subscore (Table 1) with a mean Subscore at baseline of 2.4 (95% CI: 1.7 to 3.1). Patients who did not achieve endoscopic improvement (Fig. 2A) despite ongoing anti-TNF antibody treatment with infliximab or adalimumab were enrolled. Add-on submucosal injections of GUT-1 to the ongoing anti-TNF antibody therapy induced 20% (*n* = 1/5), 60% (*n* = 3/5) and 80% (*n* = 4/5) of endoscopic improvement at week 2, 4, and 6, respectively (Fig. 2A). The endoscopic pictures of the heaviest lesion at baseline and the same lesion at weeks 2, 4 and 6 in the three corticosteroid-dependent patients (case 1, 4 and 5) are shown in Fig. 2B. At baseline, marked mucosal inflammation with multiple ulcerations and spontaneous bleeding remained present. Endoscopic findings improved within repeated GUT-1 applications from week 2, 4 to 6 (Fig. 2B).

### Efficacy in clinical responses

**Fig. 3:**
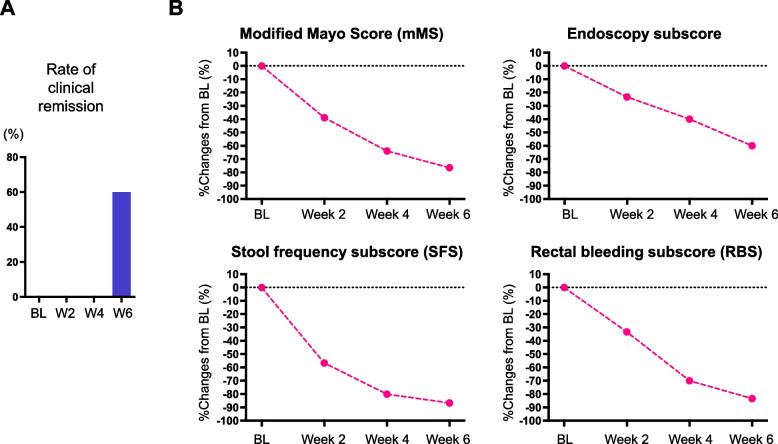
Induction of clinical remission by add-on GUT-1. **A** Rates of clinical remission at baseline (BL), week 2, 4 and 6. **B** % changes from baseline in the mean mMS, Endoscopic Subscore, SFS and RBS. Means are shown

All patients achieved clinical response at week 6 (Table 2). Rate of clinical remission at week 6 was shown by 60% (*n* = 3/5) by add-on GUT-1 treatment (Fig. 3A). The mean % change of mMS at week 2 from baseline was -38.7% (95% CI: -65.9 to -11.9) (Fig. 3B). The mean % change at week 2 from baseline of 3 mMS components were as follows; Endoscopy Subscore was -23.3% (95% CI: -51.1 to 4.4), Stool Frequency Subscore (SFS) was -56.7% (95% CI: -91.3 to -22.0) and Rectal Bleeding Subscore (RBS) was -33.3% (95% CI: -72.1 to 5.4).

At week 4, the mean % changes from baseline were as follows; mMS was -63.9% (95% CI: -69.2 to -58.7), Mayo Endoscopy Subscore was -40.0% (95% CI: -71.4 to -8.6), SFS was -80.0% (95% CI: -102.7 to -53.8) and RBS was -70.0% (95% CI: -92.7 to -47.4). At the end of induction regimen (week 6), mMS was -76.5% (95% CI: -93.8 to -59.3), Mayo Endoscopy Subscore was -60.0% (95% CI: -91.4 to -28.6), SFS was -86.7% (95% CI: -109.3 to -64.0) and RBS was -83.3% (95% CI: -112.6 to -54.1) (Fig. 3B).

### Efficacy in histological response

**Fig. 4:**
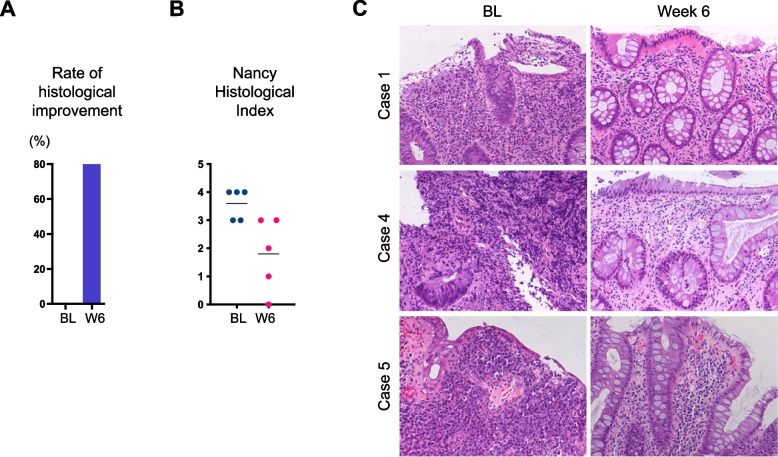
Induction of histological improvement by add-on GUT-1. **A** Rates of histological improvement at baseline (BL) and week 6. **B** Individual Nancy histological index at baseline (BL, dotted blue) and the end of induction study (week 6; W6, dotted pink). Lines indicate mean values. **C** Typical histological findings from baseline (BL, left panels) to week 6 (right panels) in 3 corticosteroid-dependent UC patients (case 1, 4 and 5) are shown. HE findings of the heaviest inflammation at week 0 and 6. Original magnification, × 200

Rate of histological improvement at week 6 was shown in 80% (*n* = 4/5) by add-on GUT-1 treatment (Fig. 4A). Add-on GUT-1 application reduced the NHI from baseline (Mean: 3.6, 95% CI: 2.9 to 4.3) to week 6 (Mean: 1.8, 95% CI: 0.2 to 3.4, Fig. 4B). Baseline mucosal histology was characterized by massive inflammatory infiltrates, loss of goblet cells, damaged epithelium, ulceration and bleeding (Fig. 4C, left panels). Add-on GUT-1 treatment reduced infiltration of lamina propria (LP) neutrophils while increased the number of goblet cells (Fig. 4C, right panels).

### Long-term observation

All patients continued to receive the same anti-TNF antibody as a standard maintenance regimen after stopping add-on GUT treatment (Fig. 1). Although case 5 did not reach endoscopic improvement at week 6, the patient later showed endoscopic improvement at week 28, and thus achieved clinical remission (Tables 2 and 3). The rate of endoscopic improvement was 100% (*n* = 5/5) and that of clinical remission was 80% (*n* = 4/5) at week 28 during the follow-up period.

**Table 3:** Period for corticosteroid free remission after achieving clinical remission

Case	Week confirming clinical remission^a^	Week confirming clinical recurrence^a^	Final observation without recurrence^a^	Corticosteroid free remission^b^
1	6	-	52	46 weeks
4	6	36	-	30 weeks
5	28	-	52	24 weeks

^a^Weeks from baseline are shown

^b^All patients did not receive corticosteroid after achieving clinical remission. Period of “Corticosteroid free-remission”, which is exploratory defined as a period from the week confirming clinical remission to the week confirming clinical recurrence or week 52 (end of the observation) in case of no recurrence is shown

Rectal bleeding was firstly observed in a patient (case 4) at week 36 and in the next patient (case 2) at week 44. Both cases were examined by endoscopy and judged as recurrence (Table 2). Recurrence-free periods after achieving clinical response by add-on GUT-1 treatment (week 6) were thus 30 weeks (case 4) and 38 weeks (case 2), respectively. Others (cases 1, 3, 5) did not show any signals of clinical recurrence for 52 weeks (Table 2), thus recurrence-free period in these 3 patients after achieving clinical response was 46 weeks. In addition, 3 corticosteroid-dependent patients (cases 1, 4, 5) received stable doses of corticosteroids at baseline and the corticosteroid doses were individually tapered during the induction therapy at the treating physicians’ discretion. All patients showed no corticosteroid exposure for at least 24 weeks after achieving clinical remission during the observation period (Table 3).

### Safety

Add-on GUT-1 was generally well tolerated. There was no observed AEs during the induction study period.

## Discussion

The participants enrolled in this IIT represented patients with moderate-to-severe active UC who did not sufficiently respond to ongoing anti-TNF antibody treatment of IFX or ADA. Add-on multiple injections of 250 nM of GUT-1 to ongoing anti-TNF antibody treatment reduced SFS at week 2, reduced RBS and Endoscopy Subscore till week 4 and induced 80% of endoscopic improvement, 100% of clinical response and 60% of clinical remission at week 6. Upon achieved clinical response at week 6 by add-on GUT-1 induction regimen, the minimum recurrence free period was 30 weeks and 60% of patients were free from recurrence for 46 weeks under continued standard maintenance regimen of anti-TNF antibody. Rapid and durable responses by add-on GUT-1 treatment is therefore suggested in the present study.

Approximately 10–40% of patients of IBD have been reported to demonstrate primary non-response to TNF antagonists [24–26]. It has also been reported that up to 50% of patients exhibited secondary loss of response (LOR) over time [24–26]. Considering the need for long-term management of IBD patients, it is a challenge to control patients with primary nonresponse and LOR to TNF antagonists. Therapeutic drug monitoring has thus been extensively investigated to manage these patients. In a histopathological point of view, intestinal fibrosis/bowel tissue remodeling is considered to be associated with nonresponse to TNF antagonists [27–29], although the precise mechanism is unexplored. In histological evaluation of UC, submucosal fibrosis was demonstrated to be directly linked with severity of mucosal inflammation and improvement of mucosal histology is hard to be achieved by conventional biological therapies including IFX and vedolizumab even after achieving endoscopic improvement [28–30]. Thus, it is of interest to investigate if anti-tissue remodeling agents might have an impact on mucosal inflammation as well as responsiveness in UC patients with TNF antagonist-nonresponse.

The mode of action of GUT-1 has been shown to reduce CHST15 mRNA and protein expression of activated fibroblast which in turn inhibits the biosynthesis of CS-E, leading to repression of submucosal fibrosis [9, 11, 15, 17]. As submucosal fibrosis leads to clinical symptoms such as loose stools, diarrhea, urgency, abdominal discomfort, pain and tenesmus [30–32], successful repression of submucosal fibrosis and thereby mucosal inflammation may contribute to improve such clinical symptoms [16]. In the present IIT, 250 nM of add-on GUT-1 application reduced SFS at week 2 after the first dosing, suggesting that improvement of stool frequency may be the first clinical sign of GUT-1’ s effectiveness. In this respect, the SFS reduced further from week 2 to 4 after the second injection of GUT-1 at week 2 in the present study. In addition, declines of RBS and Endoscopy Subscore were observed at week 4 and the Mayo Endoscopy Subscore was further reduced from week 4 to 6 after the third injection of GUT-1 at week 4. Repeated injections may contribute to these reductions, and are thus considered to be more effective to achieve clinical remission during induction therapy especially in TNF antagonist-non-responders.

Histological activity was exploratory investigated in the present IIT. A consensus of “histological improvement” is not fully established, but is defined as an over 1-point reduction from baseline by the NHI [23]. We therefore followed the current consensus and found that 80% of patients with add-on GUT-1 250 nM administration achieved histological improvement during the induction period (week 6). Most of them were attributable to reduced neutrophils infiltration. As mucosal neutrophils infiltration has recently been shown to be associated with treatment resistance as well as fibrosis [33], reduced infiltration of neutrophils is thought to be a main effect of add-on GUT-1 administration to anti-TNF antibody therapy in the present study. As CHST15-derived CS-E is shown to be a functional receptor for platelet factor 4/CXCL4 on the neutrophils for activation [34, 35], blockade of CS-E signaling may also suppress the activity of inflammatory neutrophils.

There were several limitations to the present study. First, the sample size of 5 is small. Although all patients showed clinical response at week 6, our results must be regarded as preliminary and need to be confirmed in larger clinical studies. Second, this was an open-label, single-arm design. Historical placebo data might support interpretation of the present results. Previous key studies reported that the rate of clinical remission in the placebo groups in TNF antagonist-experienced patients were 9.3% (placebo for Adalimumab), 5.4% (placebo for Vedolizumab), 5.3% (placebo for Ustekinumab), 8.2% (placebo for Tofacitinib) and 6.0% (placebo for Ozanimod) [36–42], which were less than that in TNF antagonist-naïve patients [43]. Considering the difficulty to achieve clinical remission in TNF antagonist-experienced patients, 60% and 80% of clinical remission at week 6 and week 28 respectively in the present study is thought to be relatively higher percentages. The rates of endoscopic improvement, previously termed as “mucosal healing”, in the placebo groups in TNF antagonist-experienced patients showed 31.1% (placebo for Adalimumab), 24.8% (placebo for Vedolizumab), 13.8% (placebo for Ustekinumab), 15.6% (placebo for Tofacitinib) and 11.6% (placebo for Ozanimod) [36–42], which were similar or less than that in TNF antagonist-naïve patients [44]. Likewise, 80% and 100% of endoscopic improvement at week 6 and week 28 respectively in the present study is thought to be relatively higher percentages. Third, drug trough serum concentrations and anti-drug antibodies were not measured in the present study. Thus, impact of therapeutic drug monitoring on the effect of GUT-1 is not known in the present study and should be investigated in the future.

Nevertheless, several clinical advantages are suggested in the present study. First, no GUT-1 related adverse event was reported. This would increase clinical feasibility for add-on usage to anti-TNF antibodies or other biologics in patients with immunological disorders. Second, a quick improvement of stool frequency by add-on GUT-1 application was observed. The rapid clinical signs of improvement would be beneficial especially in patients who urgently require alternative therapeutic options including surgery. Third, although exploratory, a durability of add-on GUT-1, including a potential for corticosteroid-free remission, was also shown in the present study. This would contribute to improve efficacy of conventional and developing systemic biologics in maintenance therapeutic regimen.

## Conclusions

The present study shows a potential that add-on multiple injections of GUT-1 could provide as a novel therapeutic option for additional induction therapy in patients with anti-TNF antibody-refractory UC.

## Data Availability

Individual participant data collected during the trials after de-identification are available for the present study. The requests are reviewed and approved by an independent review panel based on scientific merit. All data provided are anonymized to respect the privacy of patients who have participated in the trial, in line with applicable laws and regulations. Proposals should be directed to corresponding author at kenji-suzuki@nuhw.ac.jp. to gain access, data requestors will need to sign a data access agreement.

## Abbreviations

ADA: Adalimumab
5-ASA: 5-Aminosalicylic acid
BL: Baseline
CHST: Carbohydrate sulfotransferase
CS: Chondroitin sulfate
HE: Hematoxylin
IFX: Infliximab
IIT: Investigator-initiated trial
LOR: Loss of response
MS: Mayo Score
NHI: Nancy histological index
RBS: Rectal bleeding subscore
SFS: Stool frequency subscore
AE: Adverse event
TNF: Tumor necrosis factor
UC: Ulcerative colitis
